# Carnosine as a protective metabolic mediator in inflammatory lung injury by inhibiting macrophage infiltration and M1-like polarization

**DOI:** 10.3389/fphar.2025.1689575

**Published:** 2025-11-21

**Authors:** Lianjie Ruan, Dekai Lin, Binqin Lin, Qingqing Zhan, Lili Zheng, Dandan Lin, Yaoning Zhuang, Yiming Zeng

**Affiliations:** 1 Department of Respiratory and Critical Care Medicine, Affiliated Hospital of Putian University, Putian, Fujian, China; 2 Department of Pneumology, The Second Affiliated Hospital of Fujian Medical University, Quanzhou, Fujian, China

**Keywords:** inflammation, lung injury, carnosine, immune homeostasis, metabolomic profiling

## Abstract

**Background:**

Inflammatory lung injury is a common pathological feature of pneumonia caused by various infectious and non-infectious agents. However, metabolic regulators that can mitigate inflammation and immune cell infiltration in diverse lung injury models remain poorly understood.

**Methods:**

Using targeted metabolomic profiling of lung tissues collected on day 5 from two distinct murine models of lung inflammation—lipopolysaccharide (LPS)-induced and papain-induced—we identified carnosine as a commonly downregulated metabolite in both models. To evaluate its therapeutic potential, we administered exogenous carnosine in both models and assessed its effects on body weight, inflammatory cytokine expression, and histopathological changes.

**Results:**

Carnosine supplementation significantly improved body weight maintenance, reduced the expression of pro-inflammatory cytokines, and attenuated histological lung damage in both LPS- and papain-induced lung injury models. Flow cytometry analysis revealed that carnosine treatment markedly decreased pulmonary infiltration of macrophages and neutrophils. Multiplex immunofluorescence further demonstrated a significant reduction of macrophage accumulation in the peribronchial regions of the lung following carnosine administration. In vitro experiments using bone marrow–derived macrophages (BMDMs) confirmed that carnosine effectively suppressed LPS-induced inflammatory responses and inhibited polarization toward the M1-like macrophage phenotype.

**Conclusion:**

Our findings identify carnosine as a protective metabolic mediator in inflammatory lung injury and demonstrate its capacity to alleviate pulmonary inflammation by modulating innate immune cell recruitment and macrophage polarization. These results highlight the translational potential of carnosine as a therapeutic agent for treating inflammatory lung diseases.

## Graphical Abstract

**Figure:**
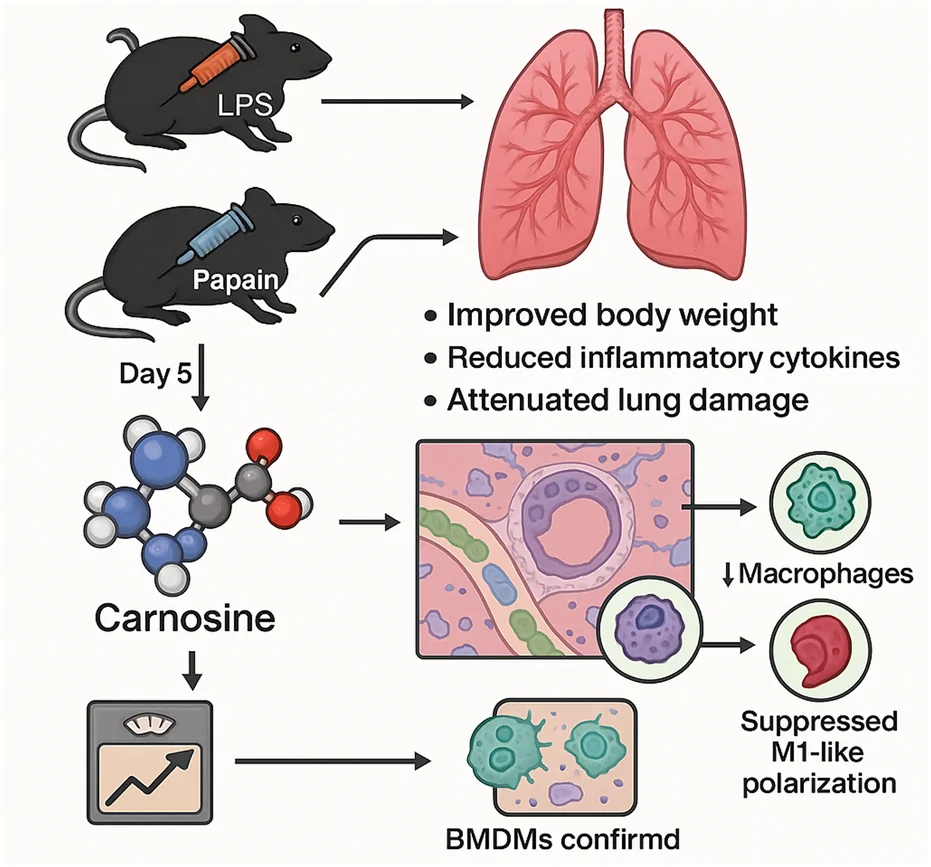

## 1 Introduction

Inflammatory lung injury is a common pathological feature observed in various pulmonary diseases, including pneumonia, acute respiratory distress syndrome (ARDS), and chronic obstructive pulmonary disease (COPD) (Park et al., 2022; Zou et al., 2023; Price and Garcia, 2024). This condition is typically characterized by excessive immune activation, cytokine release, and the infiltration of inflammatory cells—particularly neutrophils and macrophages—into lung tissue, resulting in epithelial damage, alveolar–capillary barrier disruption, and impaired gas exchange (Wang and Wang, 2023; Scozzi et al., 2022; Kolaczkowska and Kubes, 2013). Although a variety of infectious (e.g., bacterial and viral) and non-infectious (e.g., allergens and proteases) agents can trigger pulmonary inflammation, the underlying metabolic changes that modulate the immune responses in this context are not fully understood.

Emerging evidence suggests that metabolic reprogramming plays a pivotal role in regulating the immune response during pulmonary inflammation (O’Neill et al., 2016; Kelly and O’Neill, 2015; Sun et al., 2020). Metabolites not only reflect the inflammatory microenvironment but also actively shape immune cell phenotypes and functions (Zheng et al., 2025; Jung et al., 2019). However, the metabolic mediators that exert protective effects across different models of lung injury are still largely undefined. Identifying conserved, endogenous metabolic suppressors of inflammation across diverse inflammatory contexts may offer promising targets for host-directed therapies.

Carnosine, a naturally occurring dipeptide composed of β-alanine and histidine, has been implicated in antioxidative, anti-inflammatory, and cytoprotective processes in various disease models (Boldyrev et al., 2013; Saadati et al., 2024; Keshteli et al., 2022). Despite its broad biological functions, the role of carnosine in the context of lung inflammation has not been systematically evaluated. Previous studies have demonstrated that carnosine can ameliorate immune hyperactivation markers in both lipopolysaccharide (LPS)-induced and bleomycin-induced lung injury models. These findings suggest that carnosine may help mitigate excessive immune responses in preclinical models of pneumonia (Feehan et al., 2021; Tanaka et al., 2017; D’Amato et al., 2024; Babizhayev and Deyev, 2012). Recent work has examined carnosine supplementation in organs with inherently low carnosine content—most notably, the lungs—using animal models of influenza virus infection, LPS-induced acute lung injury, and pulmonary fibrosis. Among carnosine’s recognized protective actions, its antioxidant activity has gained increasing attention as a promising mechanism for the treatment of lung diseases (Saadati et al., 2024; Tanaka and Kawahara, 2020; Guney et al., 2006; Sun et al., 2017). In this study, we employed targeted metabolomic profiling in two distinct murine models of lung inflammation: one induced by LPS, a bacterial endotoxin that simulates infectious pneumonia, and the other induced by papain, a cysteine protease that models allergic and non-infectious airway injury (Izadparast et al., 2022; López et al., 2024). Our analysis revealed a consistent and significant downregulation of carnosine in both models. We then tested the therapeutic potential of carnosine supplementation and evaluated its effects on pulmonary inflammation, immune cell infiltration, and macrophage activation both *in vivo* and *in vitro*.

To explore the therapeutic potential of carnosine in inflammatory lung injury, we administered exogenous carnosine to both the LPS- and papain-induced mouse models and evaluated its effects on body weight, inflammatory cytokine production, and lung histopathology. We further investigated its impact on innate immune cell recruitment and polarization, particularly focusing on macrophage and neutrophil infiltration, along with M1 macrophage differentiation, both *in vivo* and *in vitro*. Our findings reveal that carnosine acts as a protective metabolic mediator that ameliorates lung inflammation by suppressing myeloid cell infiltration and reprogramming macrophage activation, suggesting its potential as a novel therapeutic candidate for inflammatory lung diseases.

## 2 Materials and methods

### 2.1 Materials and reagents

L-Carnosine, lipopolysaccharides, and papain were purchased from MCE (New Jersey, United States). The following reagents were also used in our study: 1640 (Gibco, Grand Island, United States), FBS (Gibco, Grand Island, United States), trypsin (Amresco, United States), EDTA (Amresco, United States), ELISA Kit for TNF-α and IL-6 (R&D Systems, Minneapolis, United States), Annexin V-FITC/PI Kit (BD, San Jose, United States), APC-conjugated anti-mouse CD8 (BioLegend, San Diego, United States), BV421-conjugated anti-mouse CD86 (BioLegend, San Diego, United States), BV711-conjugated anti-mouse CD64 (BioLegend, San Diego, United States), FITC anti-mouse MERTK Monoclonal Antibody (BioLegend, San Diego, United States), PECY7-conjugated anti-mouse CD11b (BioLegend, San Diego, United States), PE-conjugated anti-mouse CD3 (BioLegend, San Diego, United States), PEconjugated anti-mouse B220 (BioLegend, San Diego, United States), AlexaFluor® 594 anti-mouse CD326 (Ep-CAM) Antibody (BioLegend, San Diego, United States), Alexa Fluor® 647 mouse anti-Siglec-F (BioLegend, San Diego, United States), eFluor 570 mouse anti-F4/80 (BioLegend, San Diego, United States), eBioscience™ Fixable Viability Dye eFluor™ 780 (eBioscience™, San Diego, United States), DNase I (Roche, Basel, Switzerland), DTT (Merck–Roche, Basel, Switzerland), collagenase IV (Sigma-Aldrich, St. Louis, United States), Precision Count BeadsTM (BioLegend, San Diego, United States), Fixation/Permeabilization Concentrate (Invitrogen, Carlsbad, United States), PercollTM (GE Healthcare, Little Chalfont, Buckinghamshire, UK), ACK lysing buffer (Gibco, Grand Island, United States), Triton X-100 (Sangon Biotech), Tissue-Tek® O.C.T. Compound (Sakura Finetek), sucrose (Sigma-Aldrich, St. Louis, United States), and bovine serum albumin (Sigma-Aldrich, St. Louis, United States).

### 2.2 Animal model

Male C57BL mice, aged 6–8 weeks, were obtained from the Laboratory Animal Center at Fujian Medical University. All animal procedures were conducted in strict accordance with the Guide for the Care and Use of Laboratory Animals published by the U.S. National Institutes of Health (NIH Publication No. 85-23, revised 1996). Prior to experimentation, all mice were housed in a specific pathogen-free (SPF) facility under controlled environmental conditions (temperature: 24 °C ± 2 °C; humidity: 60% ± 5%) with a 12-h light/dark cycle. The animals had *ad libitum* access to standard chow and water throughout the study period.

### 2.3 Animal model of lung inflammation and tissue preparation

Male C57BL/6 mice (6–8 weeks old and weighing 20 -25 g) were housed under SPF conditions. To induce lung inflammation, the mice were anesthetized and were administered an intranasal instillation of a mixture containing LPS (5 mg/kg) and papain (10 units/mouse) dissolved in sterile PBS, with sample sizes of n = 2 and n = 3 for the two independent experiments. The LPS used in this study to induce pneumonia/acute lung injury was obtained from the *E. coli* serotype O111:B4 strain (Sigma-Aldrich), which is a commonly used strain to induce acute lung injury and pneumonia *in viv* *o*. The control mice received an equal volume of PBS. The mice were administered the mixture once a day for three consecutive days. Five days later, the mice were euthanized, and their lungs were harvested, rinsed with cold PBS, snap-frozen in liquid nitrogen, and stored at −80 °C until further analysis. The dose of 50 mg/kg body weight of L-carnosine, administered via intraperitoneal injection (I.P.) for three consecutive days, was chosen based on previous studies that had demonstrated the efficacy of similar doses in reducing inflammation and improving tissue repair in lung injury models. We started treatment one day before inducing inflammation to allow carnosine to begin exerting its protective effects before the peak of inflammatory responses. This early intervention was intended to assess carnosine’s potential as a preventive therapeutic agent rather than as a post-injury treatment, which may better mimic clinical scenarios in which early intervention is critical for managing inflammation. For cytokine measurement, lung tissues were homogenized in cold lysis buffer (containing protease inhibitors) using a mechanical homogenizer, followed by sample centrifugation at 12,000 × g for 15 min at 4 °C. The resulting supernatant was collected for ELISA.

### 2.4 Sample preparation and metabolome processing

Mice were deeply anesthetized with isoflurane at each time point; then, blood was collected from the heart apex and left on ice for 20 min. Serum was then collected by centrifugation at 12,000 g for 10 min at 4 °C and stored at −80 °C. After blood collection, the lung tissues were harvested immediately and washed with precooled PBS; excess moisture was removed, and the samples were flash-frozen in liquid nitrogen before being stored at −80 °C. Serum (thawed on ice) metabolites were extracted by adding 200 μL of methanol at −80 °C to 50 μL of the serum sample, followed by vortexing for 5 min, incubation on ice for 10 min, and centrifugation at 4 °C and 12,000 g for 15 min. To extract metabolites from tissue samples, the frozen tissue samples were first weighed (∼20 mg each sample) and ground using a homogenizer. They were then mixed with 1 mL of a 40:40:20 methanol: acetonitrile: water solution precooled to −20 °C, followed by vortexing for 5 min, incubation at −20 °C for 10 min, and centrifugation at 4 °C and 12,000 g for 15 min. D5-phenylalanine was added as the internal standard. The supernatant was then freeze-dried in a vacuum concentrator (Labconco, CentriVap Benchtop Centrifugal Vacuum Concentrator) at 4 °C, then dissolved in 50 μL of 50% (v/v, in water) acetonitrile with formic acid, and vortexed three times for 30 s, followed by centrifugation at 14,000 × g for another 10 min at 4 °C. Then, 5 µL of the supernatant was injected into the LC-MS system (A), with separate injections performed for both positive and negative ionization modes.

The area ratio values obtained from the analysis were normalized using statTarget and internal standard correction. The corrected area ratio values were used for chart presentation. MetaboAnalyst 6.0 (https://www.metaboanalyst.ca/MetaboAnalyst/ModuleView.xhtml) was used to process the metabolome data.

### 2.5 Cell viability and cytotoxicity assays

Cell viability after L-carnosine exposure was assessed using the Cell Counting Kit-8 (CCK-8, Dojindo), according to the manufacturer’s protocol. BEAS-2B cells were seeded into 96-well plates at a density of 5 × 10^5^ cells per well in 100 µL of medium and allowed to adhere overnight at 37 °C under normoxic conditions. The following day, the cells were treated with graded concentrations of L-carnosine for 48 h. CCK-8 reagent (10 µL) was then added, and the plates were incubated for an additional 3 h before measuring absorbance at 450 nm using a BioTek microplate reader. Viability was expressed as the mean absorbance from three technical replicates in three independent experiments. The half-maximal inhibitory concentration (IC_50_) of L-carnosine was calculated using Origin 8.0.

### 2.6 Annexin V-FITC/propidium iodide double-staining analysis by flow cytometry

Apoptosis was evaluated by dual Annexin V-FITC/propidium iodide (PI) staining. BEAS-2B cells were seeded into six-well plates and exposed to increasing concentrations of L-carnosine for 48 h. Following treatment, the cells were detached with 0.25% trypsin, washed, and resuspended in 1 × binding buffer at a concentration of 1 × 10^6^ cells/mL. An aliquot of the suspension (100 µL) was incubated in the dark for 15 min with 5 µL of Annexin V-FITC and 5 µL of PI. Samples were then diluted with binding buffer and analyzed by flow cytometry within 30 min. Experiments were performed in triplicate with three independent repeats.

### 2.7 Cell culture and LPS-induced inflammatory model

The human bronchial epithelial cell line BEAS-2B was cultured in BEGM™ Bronchial Epithelial Cell Growth Medium supplemented with the recommended growth factors and maintained at 37 °C in a humidified atmosphere with 5% CO_2_. To establish an *in vitro* inflammation model, BEAS-2B cells were seeded in six-well plates at a density of 1 × 10^6^ cells/well and allowed to adhere overnight. Cells were then treated with lipopolysaccharide at a final concentration of 1 μg/mL for 24 h to induce an inflammatory response. After stimulation, cell culture supernatants were collected, centrifuged at 300 *g* for 5 min to remove debris, and stored at −80 °C until cytokine quantification.

### 2.8 Hematoxylin and eosin staining

Pathological staining of paraffin-embedded tissue sections was performed using standard histological protocols. Briefly, tissue sections were deparaffinized and rehydrated through a graded series of xylene and ethanol. Hematoxylin and eosin (H&E) staining was performed to assess the overall intestinal architecture. For Gram staining, sections were sequentially incubated with crystal violet, Gram’s iodine, and safranin, followed by decolorization with a solvent-based decolorizer and subsequent treatment with picric acid–acetone. Following final dehydration through ethanol and xylene, the sections were cover-slipped using Fluoromount Aqueous Mounting Medium (Sigma-Aldrich, Cat# F4680). Images were captured using an upright wide-field Nikon Eclipse 90i microscope. A histopathological evaluation was performed on H&E-stained lung tissue sections according to established protocols (Lee et al., 2020; Dietert et al., 2017). All sections were coded to blind the evaluators to the experimental groups, and they were examined by trained histopathologists. A histological scoring system, detailed in Supplementary Table S1, was applied to assess the severity of lung injury. Briefly, each section was first scanned at low magnification (4 ×) to determine the extent of tissue damage and was assigned an overall score ranging from 0 (normal morphology) to 3 (most diffuse tissue lesions). Subsequently, inflammatory lesions in the bronchiolar, alveolar, and vascular compartments were assessed individually, with a score from 0 to 3 assigned to each category. The maximum cumulative score for a severely injured lung tissue sample was 27.

### 2.9 Immunofluorescence staining of lung tissue

After C57BL/6 mice were induced with LPS and papain via intranasal administration, the mice were perfused with 4% PFA, and lung tissue was collected, fixed in 4% PFA (diluted with PBS at a ratio of 1:2) at 4° for 24 h, dehydrated with 30% sucrose, and then prepared as 20-μm slices after OCT embedding. The frozen sections were rehydrated with phosphate-buffered saline (PBS) and then blocked with PBS containing 1% normal mouse serum, 1% bovine serum albumin, and 0.3% Triton X-100, followed by staining with directly conjugated antibodies for 24 h at room temperature in a dark, humidified chamber. After staining, images were acquired on a Leica TCS SP8 Confocal Microscope. Images were analyzed using Imaris software (Bitplane).

### 2.10 Flow cytometry

Single-cell suspensions were obtained from a lung section by digestion with collagenase IV (0.25 mg·mL^−1^) at 37 °C for 30 min, followed by filtration through a 70-μm cell strainer and red blood cell (RBC) lysis using RBC lysis buffer (BioLegend) for 2 min at room temperature. For flow cytometry or fluorescence-activated cell sorting (FACS), the cells were stained with FACS buffer (phosphate-buffered saline supplemented with 2% bovine serum albumin and 5 mM EDTA) and monoclonal antibodies. The cell suspension was stained using fixable viability violet dye Zombie Red or Fixable Viability Dye eFluor™ 780 (Invitrogen) for 30 min at 4 °C, which was followed by blocking of Fc-receptors with CD16/32 (2.4G2, BD Biosciences) for 15 min at 4 °C. For FACS, cells were prepared and stained as described.

### 2.11 Statistical analysis

Data are expressed as the mean ± SD. Pairwise comparisons were performed using two-tailed unpaired Student’s t-tests in GraphPad Prism v8.0. Statistical significance was set at *p* < 0.05, with *p* < 0.01 being considered highly significant.

## 3 Results

### 3.1 Untargeted metabolomic profiling of serum reveals potential biomarkers for pneumonia

**FIGURE 1:**
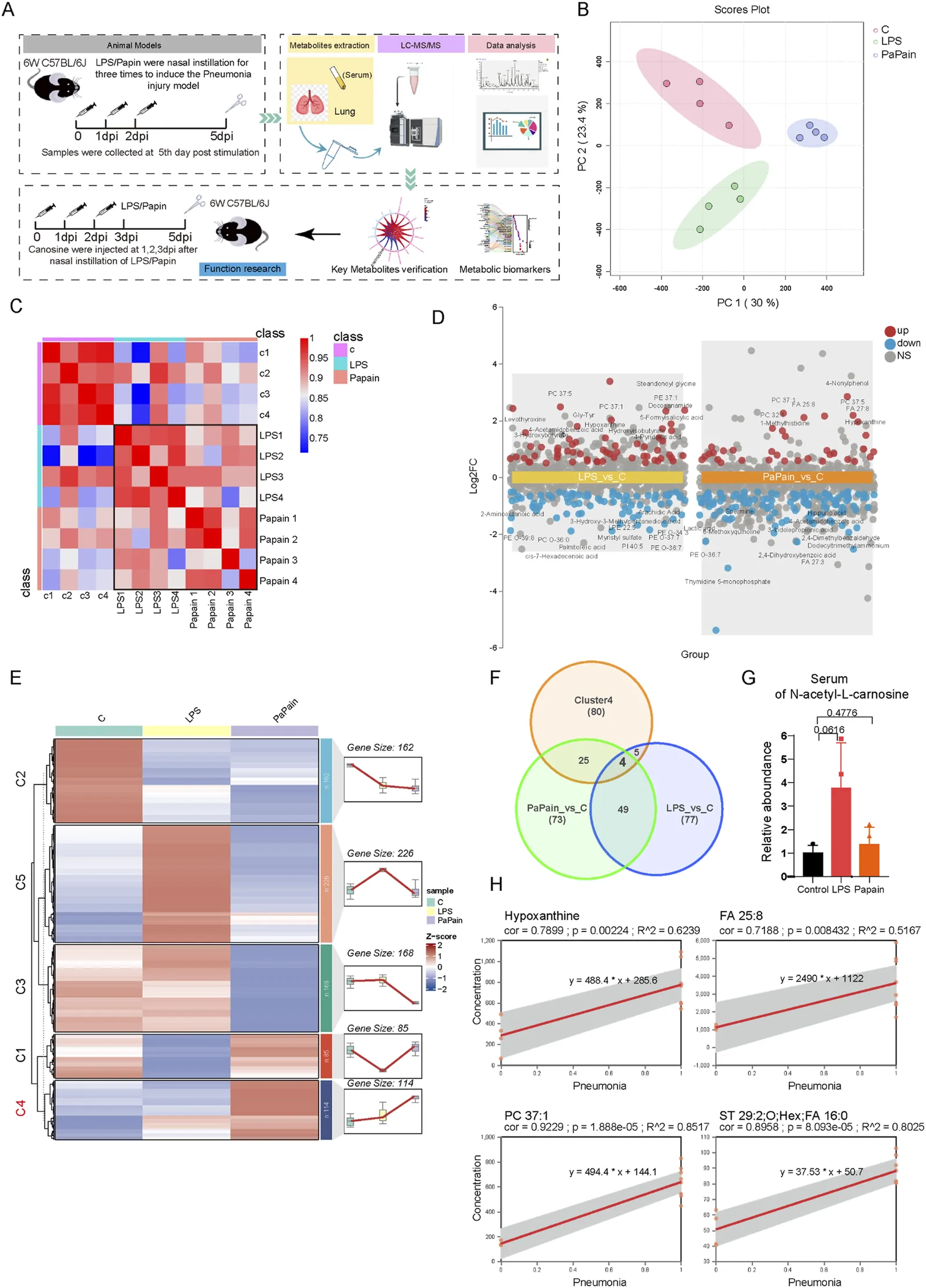
Untargeted Metabolomic Profiling of Serum Reveals Potential Biomarkers for Pneumonia. **(A)** Schematic diagram of the study design. **(B)** Principal component analysis of serum metabolomics, n=4. **(C)** Correlation analysis of the serum metabolome. **(D)** Multi-group volcano plots of altered metabolites in LPS and Papain group compared to control groups (|Log2FC|>0.5, P-value<0.05). **(E)** K-means based classification of serum metabolites among three groups for metabolic trajectory analysis. **(F)** Venn diagram of the intersection of differential metabolites in LPS_vs_C and Papain_vs_C and cluster4 metabolites. **(G)** Relative abundunce of N-Acetyl-L-Carnosine (a procedure of Carnosine) in the serum. **(H)** Linear regression models of core metabolites in the serum of pneumonia group (LPS and Papain treatment groups defined as 1) and control group (defined as 0).

To systematically elucidate metabolic reprogramming in pneumonia, we established two murine models of pneumonia using LPS and papain. Lung tissues and serum were collected on day 5 post-induction for metabolomic profiling to identify metabolic vulnerabilities associated with acute pneumonia. Figure 1A shows the study design and pattern. To identify pneumonia-associated metabolic biomarkers, untargeted metabolomics was conducted on the serum, and a total of 755 polar metabolites and lipids were detected and identified (Supplementary Table S2 ). Principal component analysis (PCA) confirmed significant metabolic divergence between the pneumonia groups and healthy controls (Figure 1B). Conversely, correlation analysis showed that the metabolic correlation co-effects were higher in the serum of both LPS- and papain-induced pneumonia groups than in the control group, indicating common systemic metabolic alterations in both models (Figure 1C). Additionally, differential analysis revealed 135 dysregulated serum metabolites in LPS-induced pneumonia and 151 in papain-induced pneumonia, respectively (Figure 1D; |Log2FC|≥0.5, *p*-value <0.05). K-means-based clustering was carried out to further characterize the serum metabolic characteristics of LPS- and papain-induced pneumonia, identifying cluster 4 metabolites as consistently upregulated in both pneumonia models (Figure 1E). Then, cluster 4 metabolites were overlapped with altered metabolites in both the LPS and papain groups, and four shared elevated candidates were defined (Figure 1F). Linear regression was conducted to verify the relationship between the four metabolites and diseases; FA (25:8), PC (37:1), and hypoxanthine showed a significantly positive correlation with pneumonia severity (Figure 1H), suggesting that these metabolites act as potential diagnostic biomarkers for pneumonia. Unfortunately, we did not detect carnosine in the untargeted serum metabolomics analysis. However, we identified N-acetyl-L-carnosine, a precursor of carnosine, which showed an increasing trend in the serum (Figure 1G). Interestingly, it was not detected in the lung tissue. This suggests that under conditions of acute lung injury or pulmonary inflammation, the body may compensatorily increase N-acetyl-L-carnosine—a precursor form that is less susceptible to enzymatic degradation—to participate in a systemic anti-inflammatory response. However, it appears that this compound does not subsequently accumulate in the lung tissue, which is a phenomenon that warrants further investigation. In summary, untargeted metabolomics analysis of serum revealed strong metabolic correlations between LPS- and papain-induced pneumonia, identifying serum biomarkers associated with pneumonia.

### 3.2 Targeted metabolomics reveals metabolic reprogramming in LPS- and papain-induced pneumonia

**FIGURE 2:**
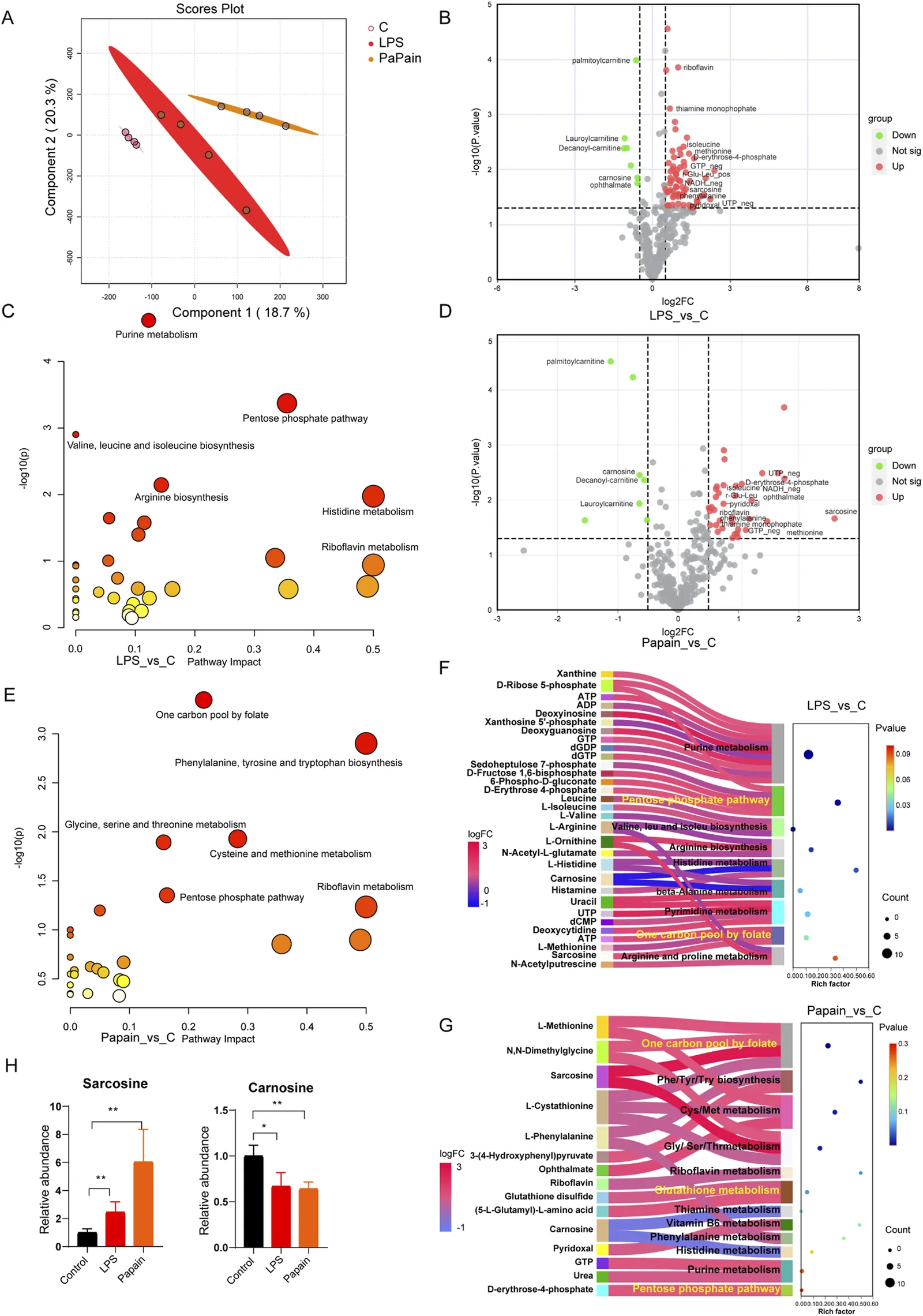
Targeted metabolomics reveals metabolic reprogramming in LPS- and papain-induced pneumonia. **(A)** Principal component analysis of lung metabolomics; n = 4. **(B)** Volcano plot displaying differential metabolites of LPS compared to the control group (LPS_vs._C, |Log2FC ≥ 0.5|, *p*-value<0.05). **(C)** KEGG pathway of differential metabolites of LPS_vs._C groups. **(D)** Volcano plot displaying the differential metabolites of papain compared to those of the control group (Papain_vs._C, |Log2FC ≥ 0.5|, *p*-value<0.05). **(E)** KEGG pathway of differential metabolites of Papain_vs._C groups. **(F, G)** Sankey diagram mapping for differential metabolites and enriched pathway in LPS_vs._C and Papain_vs._C groups. **(H)** Relative abundance of sarcosine and carnosine in the lungs.

Targeted metabolomics was performed on lung tissues, and 324 polar metabolites were detected (Supplementary Table S1). PCA revealed distinct metabolic profiles in both the LPS and papain groups compared to those in controls (Figure 2A). Differential analysis (|Log_2_FC| ≥ 0.5, *p* < 0.05) identified 72 significantly altered metabolites in LPS-induced pneumonia and 42 in papain-induced pneumonia (Figures 2B, D). Furthermore, KEGG pathway enrichment analysis demonstrated significant enrichment of purine metabolism, valine, leucine, and isoleucine biosynthesis, arginine biosynthesis, the pentose phosphate pathway (PPP), and histidine metabolism in LPS-induced pneumonia, while papain-induced pneumonia primarily enriched the one-carbon pool by folate, alanine aspartate, and glutamate metabolism, glycine, serine, and threonine metabolism, cysteine and methionine metabolism, and the PPP (Figures 2C, E). To describe the detailed metabolic changes, Sankey diagram mapping was performed, covering the majority of the metabolites within the enriched pathways. Notably, upregulated metabolites related to purine metabolism, the PPP, and one-carbon metabolism, which supply macromolecular precursors for cell proliferation, are strongly correlated with the activation of immune cell proliferation. Additionally, elevated levels of D-erythrose-4-phosphate in both models indicated increased oxidative PPP flux. Sarcosine, a metabolite involved in ATP synthesis, was significantly upregulated, likely supporting the heightened energy requirements of proliferating cells. Importantly, carnosine was consistently downregulated in both pneumonia models as it is mechanistically linked to histidine and thiamine metabolism (Figures 2F, G), which suggests that carnosine may serve as a critical pneumonia-associated metabolite. Carnosine, which has been demonstrated in previous studies to exert direct antioxidant effects, is primarily synthesized in muscle tissue. The observed depletion of carnosine likely reflects its rapid consumption due to redox imbalance in the inflamed lung, coupled with insufficient replenishment from remote synthesis sites (Figure 2H).

### 3.3 Metabolic commonalities and differences between the two pneumonia models

**FIGURE 3:**
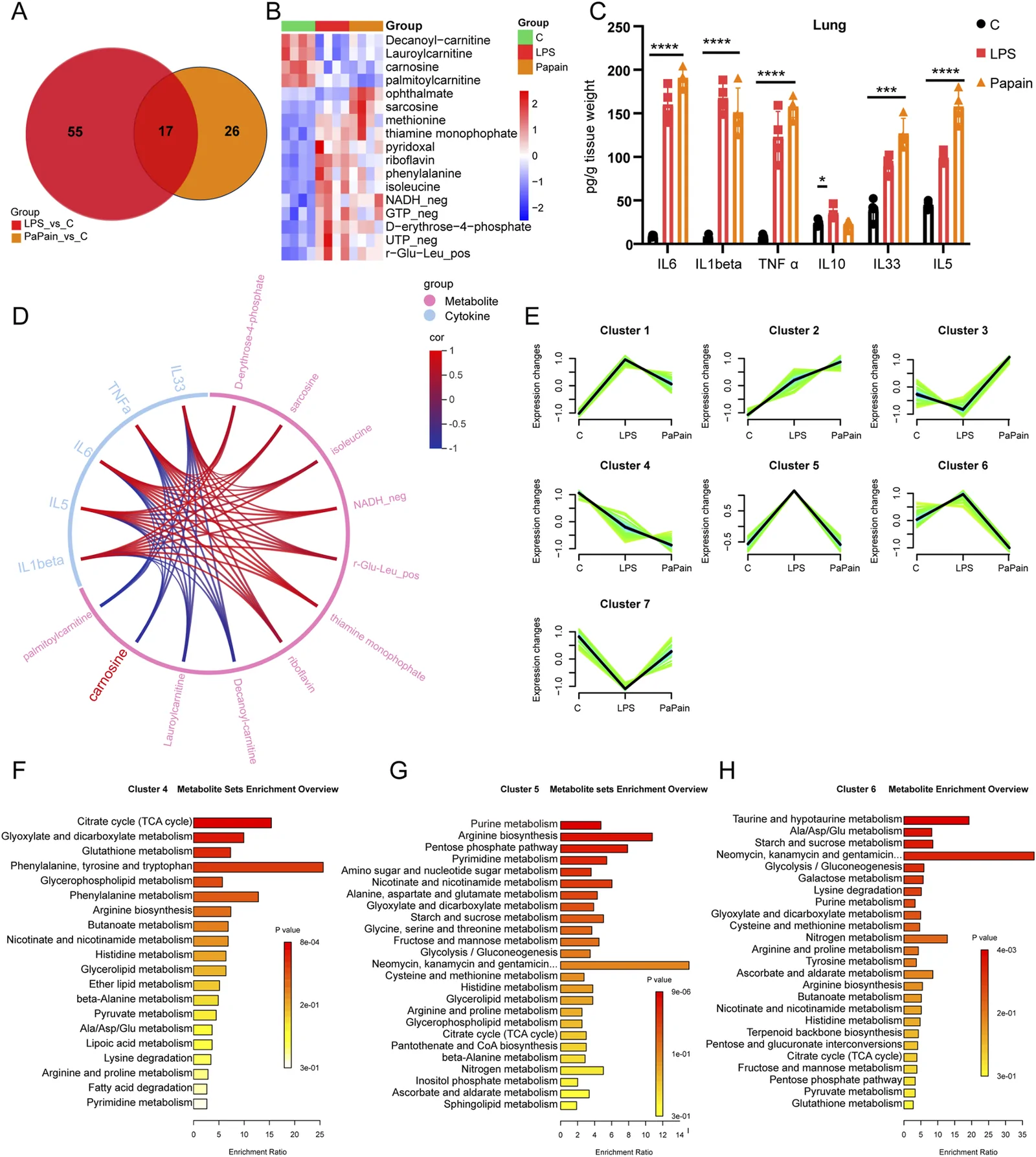
Metabolic commonalities and differences between the two pneumonia models. **(A)** Venn diagram of differential metabolites in the two pneumonia models. **(B)** Heatmap of overlapping metabolites in the pneumonia models. **(C)** ELISA measurements showing the expression of pro-inflammatory cytokines in the lung tissue after LPS or papain stimulation; n = 4/group (data are shown as the mean ± SD; *p < 0.05, **p < 0.01, and ***p < 0.001). **(D)** Chordal graph of the correlation between metabolite levels and cytokines in the pneumonia models. **(E)** Mfuzz-based clustering of metabolites in the lungs, distinguishing pneumonia-specific metabolic patterns. **(F–H)** KEGG pathway analysis of cluster 4 **(F)**, cluster 5 **(G)**, and cluster 6 **(H)** metabolites.

To compare metabolic features between the models, Venn analysis was conducted to compare the metabolic features between the two pneumonia models, revealing 17 shared differentially abundant metabolites with consistent directional changes in both models (Figures 3A, B). Conversely, pro-inflammatory cytokines (IL-6, IL-1β, TNF-α, IL-33, and IL-5) were significantly elevated in both models (Figure 3C). Spearman correlation analysis showed negative correlations between cytokine levels and a few metabolites, including palmitoylcarnitine, decanoylcarnitine, lauroylcarnitine, and carnosine (Figure 3D). The downregulation of acylcarnitines, which are involved in intracellular fatty acid oxidation, suggests impaired fatty acid oxidation and mitochondrial dysfunction. Carnosine, an endogenous antioxidant, exhibited a consistently reduced level in both models that correlated negatively with pro-inflammatory cytokine levels, suggesting its critical role in modulating pulmonary immune homeostasis during pneumonia. Mfuzz-based metabolic trajectory analysis of all lung metabolites defined seven clusters (Figure 3E). Furthermore, cluster 4 metabolites (significantly decreased in both models) were enriched in the TCA cycle and glutathione metabolism, reflecting the heightened demands for antioxidants and energy substrates; metabolites in cluster 5, which were specifically increased in LPS-induced pneumonia, were mainly enriched in purine metabolism, arginine biosynthesis, the PPP, and pyrimidine metabolism, indicating the robust immune-cell activation in LPS-induced pneumonia; metabolites in cluster 6 (decreased in the papain group uniquely) were enriched in taurine and hypotaurine metabolism and alanine aspartate and glutamate metabolism, implying systemic dysregulation of antioxidant defense mechanisms (Figures 3F–H). These findings collectively indicate dysregulated antioxidant metabolism in pneumonia, positioning carnosine supplementation as a potential therapeutic strategy.

### 3.4 L-Carnosine attenuates inflammation and immune cell infiltration in mouse models of acute lung injury

**FIGURE 4:**
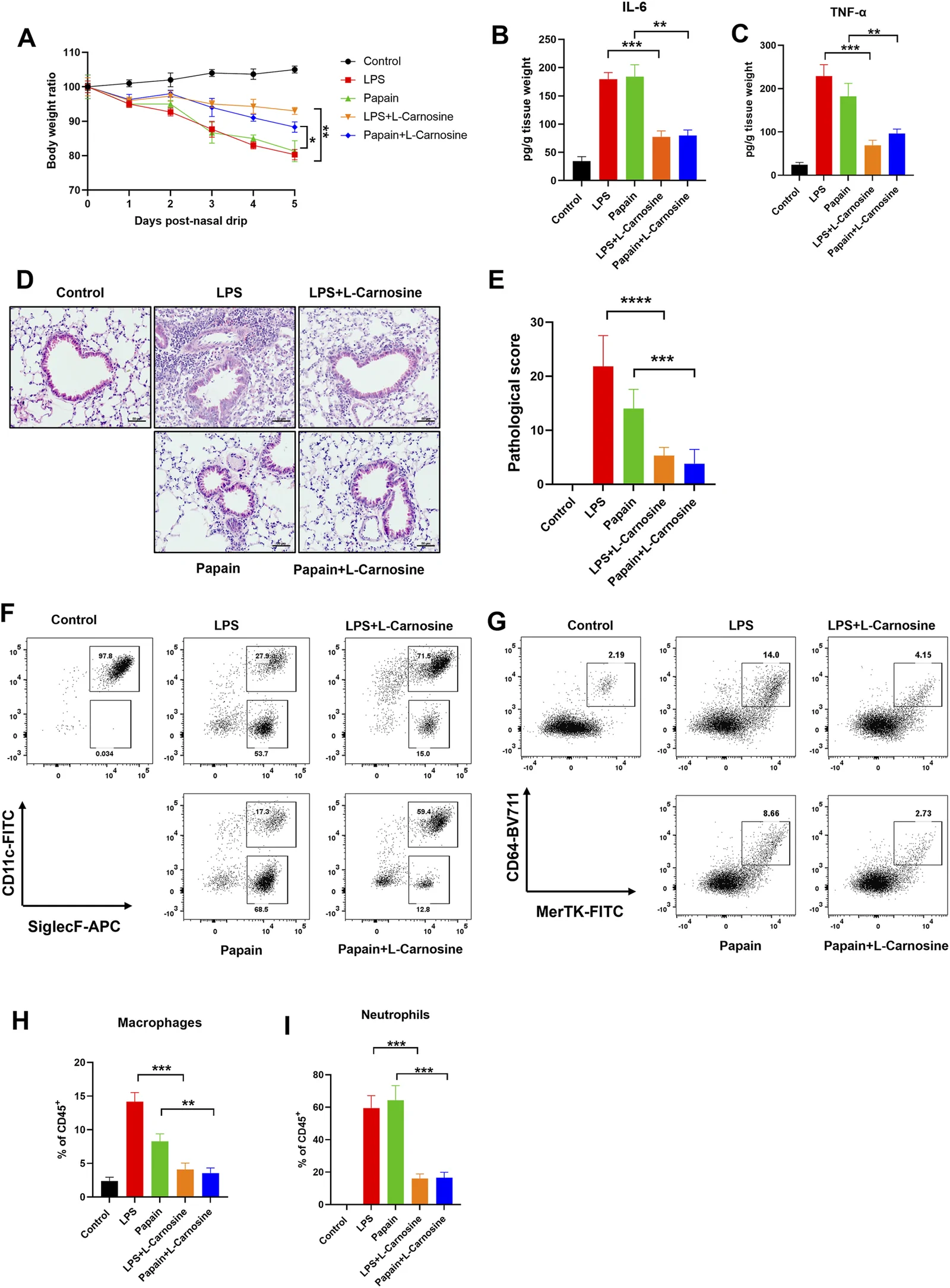
L-Carnosine attenuates inflammation and immune cell infiltration in mouse models of acute lung injury. **(A)** Body weight changes in mice subjected to LPS- or papain-induced lung injury with or without L-carnosine pretreatment (500 mg/kg, intraperitoneally, once daily for 5 days). **(B, C)** ELISA analysis of IL-6 **(B)** and TNF-α **(C)** levels in lung tissue homogenates. **(D, E)** Representative H&E-stained lung tissue sections showing histopathological changes and histopathological evaluation **(E)** in each group. **(F, G)** Flow cytometry plots showing the proportions of CD11b^+^F4/80^+^ macrophages in digested lung tissue **(F)** and CD11b^+^Ly6G^+^ neutrophils in the bronchoalveolar lavage fluid **(G)**. **(H, I)** Quantification of macrophage **(H)** and neutrophil **(I)** infiltration in each treatment group. Data are presented as the mean ± SD (n = 5 mice/group). Statistical significance was calculated by a paired, two-tailed Student’s t-test. *p < 0.05, **p < 0.01, and ***p < 0.001 vs. the model group.

To evaluate the therapeutic effect of L-carnosine on acute inflammatory lung injury *in vivo*, we used two mouse models of lung inflammation: LPS- and papain-induced acute lung injury models. Mice were pretreated with L-carnosine (500 mg/kg, intraperitoneally) 1 day prior to model induction and received continuous daily administration for five consecutive days. Body-weight monitoring revealed that both LPS and papain treatment caused significant weight loss, which was markedly alleviated by L-carnosine pretreatment (Figure 4A), indicating a systemic protective effect. Next, we assessed pulmonary inflammation by quantifying IL-6 and TNF-α levels in lung tissue homogenates. As shown in Figures 4B, C, L-carnosine treatment significantly reduced IL-6 and TNF-α production compared with the model groups, with IL-6 decreased by ∼2.3-fold in both models and TNF-α decreased by ∼3.3-fold in the LPS model and ∼1.8-fold in the papain model. Histological analysis of lung sections further confirmed that L-carnosine substantially ameliorated the pathological features of lung injury, including alveolar wall thickening, inflammatory cell infiltration, and alveolar collapse (Figures 4D, E). To investigate immune cell infiltration in the lungs, flow cytometry was performed on isolated pulmonary tissues. In both acute lung injury models, treatment with L-carnosine significantly reduced the proportion of CD11b^+^F4/80^+^ macrophages (Figure 4F) and SiglecF^+^CD11c^−^ eosinophils in the bronchoalveolar lavage fluid (Figure 4G). Quantitative analysis further confirmed a marked decrease in macrophage (Figure 4H) and eosinophil (Figure 4I) infiltration following L-carnosine administration, with macrophages reduced by ∼3.4-fold in the LPS model and ∼2.3-fold in the papain model and eosinophils reduced by ∼3.7-fold (LPS) and ∼3.9-fold (papain). Collectively, these findings indicate that L-carnosine alleviates acute lung injury by suppressing the production of pro-inflammatory cytokines and limiting the recruitment of myeloid immune cells into lung tissue.

### 3.5 L-Carnosine selectively reduces pulmonary infiltration of pro-inflammatory macrophages in acute lung injury models

**FIGURE 5:**
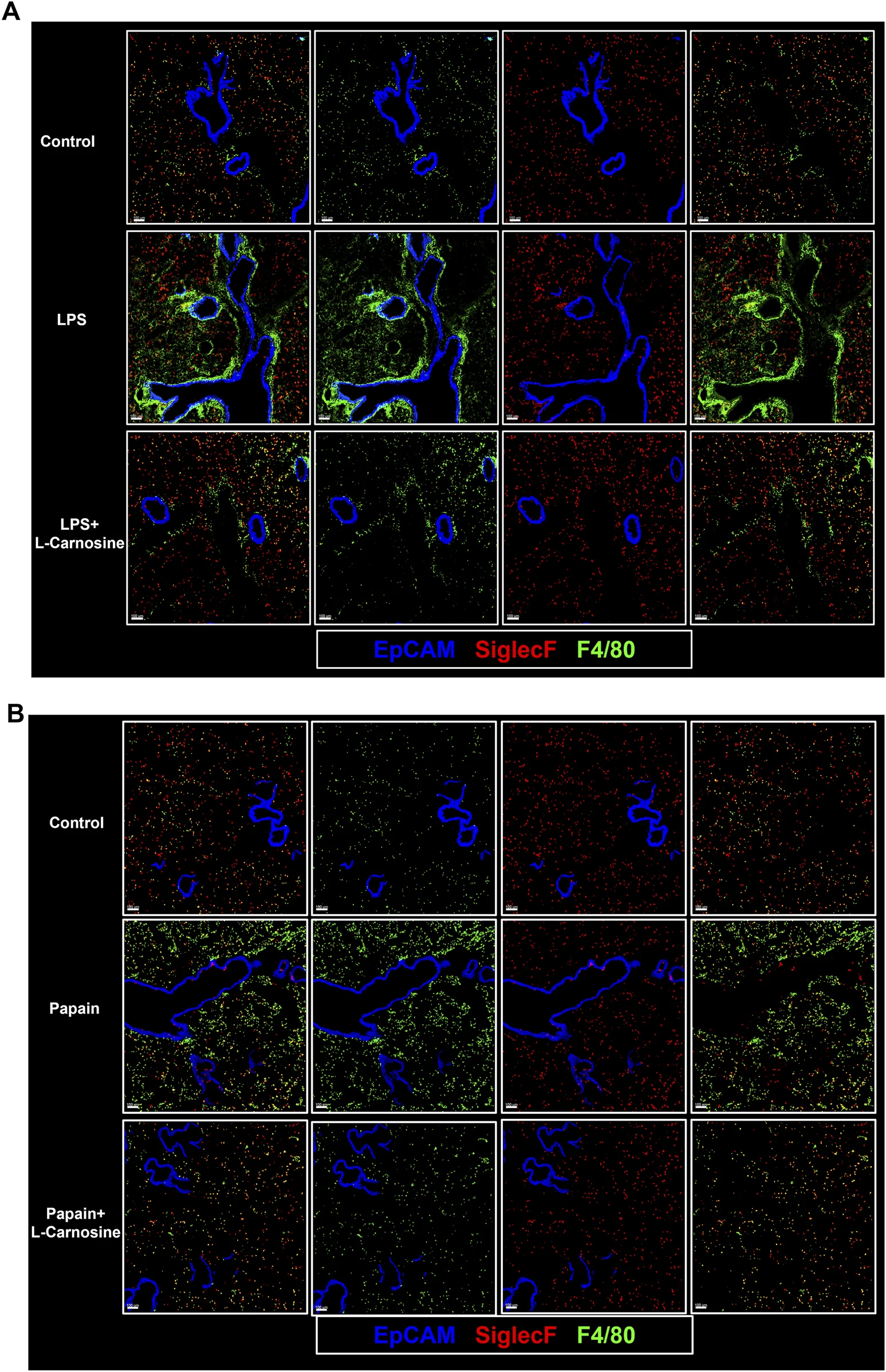
L-Carnosine selectively reduces pulmonary infiltration of pro-inflammatory macrophages in acute lung injury models. **(A)** Representative multiplex immunofluorescence images of lung tissue sections from the LPS-induced acute lung injury model, showing decreased infiltration and peribronchial accumulation of F4/80^+^Siglec-F^−^ macrophages following L-carnosine treatment. **(B)** Similar reduction in F4/80^+^Siglec-F^−^ macrophages observed in the papain-induced lung injury model upon L-carnosine administration.

To further validate the protective effect of L-carnosine on pulmonary inflammation at the cellular level, we performed multiplex immunofluorescence staining of lung tissue sections from both the LPS- and papain-induced acute lung injury models. F4/80 and Siglec-F were used to distinguish infiltrating macrophage subsets and eosinophils, respectively. In the LPS-induced model, we observed a marked increase in F4/80^+^Siglec-F^−^ macrophages, which were predominantly distributed around the peribronchial and perivascular regions. However, L-carnosine treatment substantially reduced the infiltration and peribronchial clustering of these pro-inflammatory macrophages (Figure 5A). A similar pattern was confirmed in the papain-induced lung injury model, with L-carnosine significantly limiting F4/80^+^Siglec-F^−^ macrophage accumulation in the lung tissue (Figure 5B). In contrast, the infiltration and spatial localization of Siglec-F^+^ eosinophils appeared largely unaffected by L-carnosine intervention in both models. These results suggest that L-carnosine selectively suppresses the recruitment of inflammatory macrophages without altering eosinophil trafficking, highlighting its immunomodulatory specificity in the inflammatory lung microenvironment.

### 3.6 L-Carnosine mitigates LPS-induced apoptosis and inflammatory responses in human bronchial epithelial cells

**FIGURE 6:**
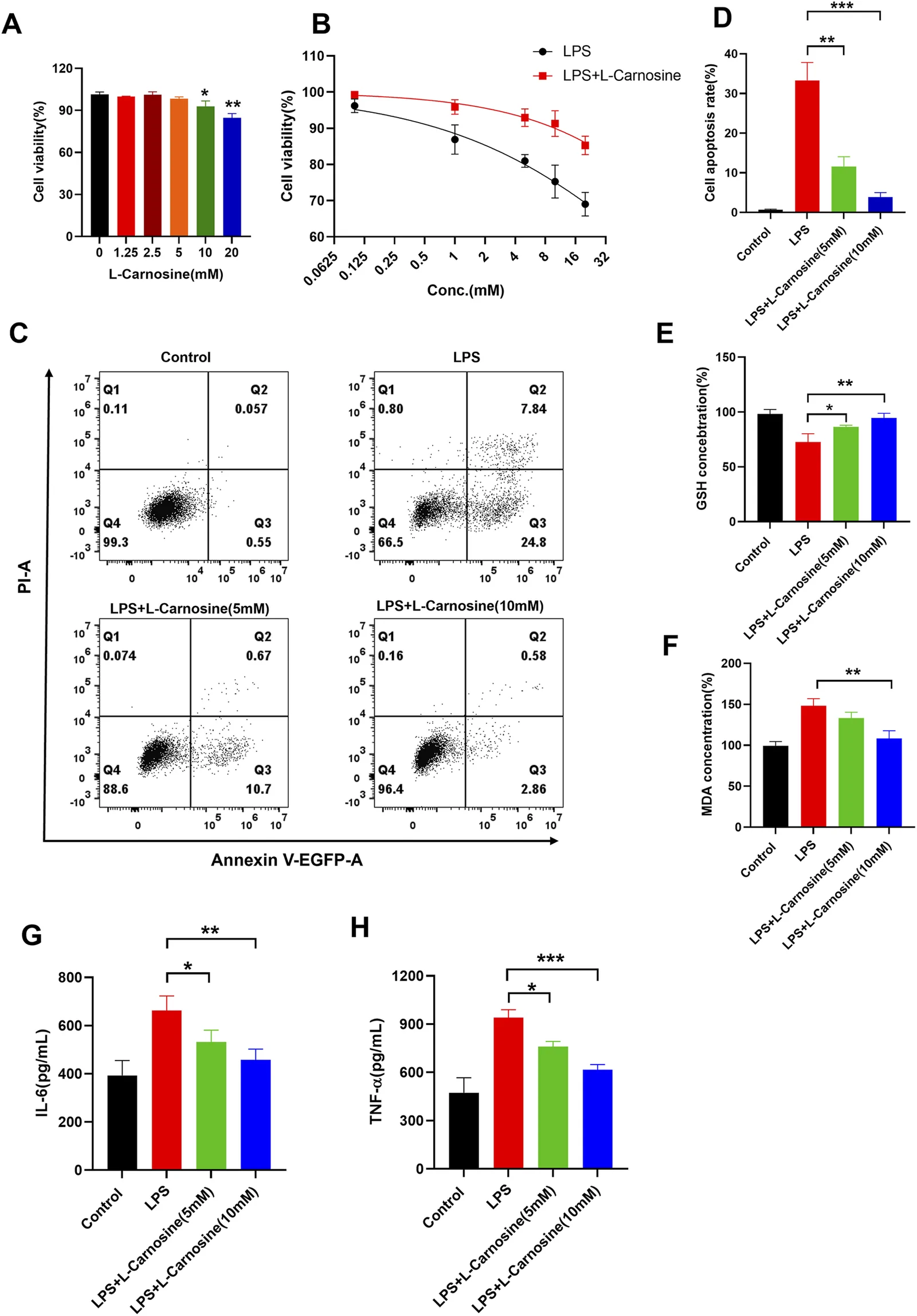
L-Carnosine mitigates LPS-induced apoptosis and inflammatory responses in human bronchial epithelial cells. **(A)** A CCK-8 assay was used to evaluate the cytotoxicity of L-carnosine in BEAS-2B cells. L-Carnosine did not significantly affect cell viability at concentrations up to 5 mM, whereas concentrations ≥10 mM significantly reduced viability. **(B)** BEAS-2B cells were co-treated with LPS and 5 mM L-carnosine. L-Carnosine significantly improved cell viability across varying concentrations of LPS. **(C)** Flow cytometric analysis of apoptosis in BEAS-2B cells treated with LPS in the presence or absence of L-carnosine. **(D)** Quantitative analysis of the apoptotic cell populations from **(C)** indicates that L-carnosine reduces LPS-induced apoptosis in a dose-dependent manner. **(E, F)** Intracellular glutathione (GSH) levels and malondialdehyde (MDA) production were measured to assess oxidative stress. L-Carnosine restored GSH levels and reduced MDA accumulation following LPS exposure. **(G, H)** ELISA analysis showed that L-carnosine markedly inhibited LPS-induced secretion of the pro-inflammatory cytokines IL-6 and TNF-α. Statistical significance was calculated by a paired, two-tailed Student’s t-test. Data are presented as the mean ± SD (n = 3); *p < 0.05, **p < 0.01, and ***p < 0.001.

To determine the optimal concentration of L-carnosine for cell-based assays, we first examined its cytotoxicity profile in BEAS-2B cells. BEAS-2B, a human bronchial epithelial cell line, is commonly used in studies of pulmonary inflammation and toxicity due to its ability to closely replicate the characteristics of human airway epithelial cells. The BEAS-2B cells retain many characteristics of primary bronchial epithelial cells, including their response to inflammatory stimuli such as LPS and papain, making them a relevant model for studying lung inflammation *in vitro*. CCK-8 analysis revealed that L-carnosine had no significant inhibitory effect on cell viability at concentrations up to 5 mM, while concentrations ≥10 mM markedly suppressed proliferation (Figure 6A). Therefore, a 5 mM concentration was selected for subsequent experiments. Next, we investigated whether L-carnosine could protect BEAS-2B cells from LPS-induced injury. Co-treatment with 5 mM L-carnosine significantly improved cell viability in the presence of various concentrations of LPS (Figure 6B). Furthermore, flow cytometric analysis showed that L-carnosine reduced LPS-induced apoptosis in a dose-dependent manner (Figure 6C), as confirmed by the quantitative data (Figure 6D). We further explored the impact of L-carnosine on oxidative stress markers. LPS exposure led to reduced intracellular GSH levels and elevated MDA production, both of which were significantly reversed by L-carnosine treatment (Figures 6E, F). Finally, ELISA analysis showed that L-carnosine markedly decreased the secretion of the pro-inflammatory cytokines IL-6 and TNF-α following LPS stimulation (Figures 6G, H), indicating an anti-inflammatory effect.

### 3.7 L-Carnosine inhibits LPS-induced inflammatory responses and M1 polarization in mouse BMDMs

**FIGURE 7:**
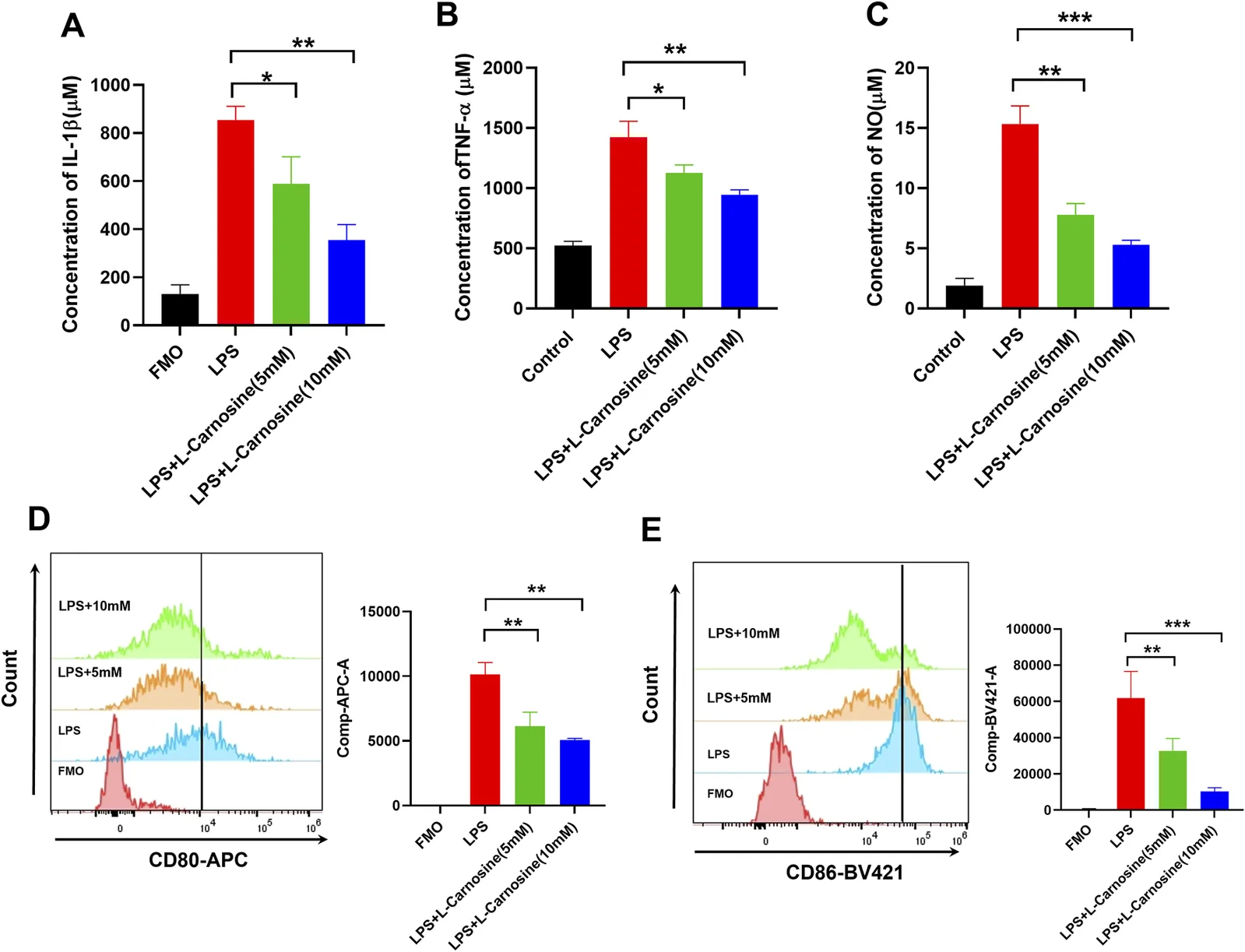
L-Carnosine inhibits LPS-induced inflammatory responses and M1 polarization in mouse BMDMs. **(A, B)** ELISA quantification of IL-1β and TNF-α levels in BMDM culture supernatants following 24 h of LPS stimulation with or without 1 h pretreatment of L-carnosine at the indicated concentrations. **(C)** Nitric oxide production, as measured by the Griess assay under the same treatment conditions. **(D, E)** Flow cytometric analysis of M1 macrophage markers CD80 and CD86 in BMDMs treated as above. Data are presented as the mean ± SD (n = 3). Statistical significance was calculated by a paired, two-tailed Student’s t-test; *p < 0.05, **p < 0.01, and ***p < 0.001 vs. LPS alone.

To investigate the anti-inflammatory properties of L-carnosine in LPS-activated macrophages, primary mouse bone marrow-derived macrophages (BMDMs) were pretreated with increasing concentrations of L-carnosine (5 and 10 mM) for 1 h prior to stimulation with LPS (100 ng/mL) for 24 h. ELISA analysis of the culture supernatants demonstrated that L-carnosine markedly and dose-dependently attenuated the release of IL-1β (Figure 7A) and TNF-α (Figure 7B) relative to the LPS-only controls. Consistently, the Griess assay revealed that L-carnosine significantly inhibited LPS-induced nitric oxide production (Figure 7C). To further investigate macrophage polarization, flow cytometry was performed to assess the surface expression of M1 markers. LPS stimulation significantly increased the mean fluorescence intensity (MFI) of CD80 and CD86 on BMDMs, whereas L-carnosine pretreatment led to a dose-dependent reduction in the MFI of both markers (Figures 7D, E). These findings suggest that L-carnosine effectively suppresses LPS-induced pro-inflammatory responses and inhibits M1-type polarization of macrophages.

## 4 Discussion

Inflammatory lung injury is a hallmark pathological feature of pneumonia caused by diverse infectious and non-infectious stimuli, including bacterial endotoxins, allergens, and environmental irritants (Gould et al., 2025; Li et al., 2025). These conditions are often driven by excessive immune activation and myeloid cell infiltration, resulting in epithelial damage, alveolar–capillary barrier disruption, and impaired gas exchange (Kolaczkowska and Kubes, 2013; Zhang et al., 2024; Wang et al., 2024). Despite extensive studies on immune mechanisms, the metabolic regulators that can effectively mitigate lung inflammation across diverse pathological contexts remain largely undefined.

In this study, we utilized targeted metabolomic profiling to investigate common metabolic alterations in two mechanistically distinct murine models of lung injury—LPS- and papain-induced inflammation. Remarkably, carnosine, a naturally occurring dipeptide composed of β-alanine and L-histidine, was identified as a consistently downregulated metabolite in both models (Gasmi et al., 2025). These findings suggest that carnosine depletion may be a metabolic signature of acute lung inflammation, regardless of the upstream trigger. Functionally, we demonstrated that exogenous administration of carnosine exerted broad protective effects in both models. Carnosine-treated mice exhibited improved weight maintenance, reduced histopathological lung injury, and significantly decreased the expression of key pro-inflammatory cytokines, including TNF-α and IL-6. These effects were accompanied by marked reductions in pulmonary infiltration of neutrophils and macrophages, as confirmed by flow cytometry and multiplex immunofluorescence. In particular, the reduction of macrophage accumulation around the bronchial regions underscores the spatial specificity of carnosine’s anti-inflammatory action.

Mechanistically, our *in vitro* data revealed that carnosine not only suppressed LPS-induced cytokine production in BMDMs but also inhibited their polarization toward the M1-like pro-inflammatory phenotype, which is known to exacerbate lung tissue damage (Murray, 2017; Li et al., 2018). This aligns with previous reports indicating that carnosine possesses anti-inflammatory and antioxidant properties in models of cardiovascular disease and neuroinflammation (Boldyrev et al., 2013; Efthymakis and Neri, 2022; Yan et al., 2024; Feehan et al., 2022; Wu, 2020; Tsai et al., 2010). Our results extend these observations to pulmonary inflammation, highlighting carnosine as a metabolically derived immunomodulator capable of reprogramming innate immune responses.

The strength of our findings lies in the use of two distinct injury models: LPS, which induces Toll-like receptor 4 (TLR4)-mediated acute inflammation mimicking bacterial pneumonia, and papain, which simulates protease-mediated epithelial injury and allergic-type inflammation. The consistent efficacy of carnosine across these models supports its broad-spectrum anti-inflammatory potential in lung injury, independent of the initiating insult. From a translational perspective, carnosine is an endogenous, non-toxic compound with a well-characterized safety profile and has already been investigated as a dietary supplement in clinical contexts (Swietach et al., 2024; Kabthymer et al., 2025). Our data suggest that carnosine or its analogs could be repurposed or optimized as therapeutic agents for inflammatory lung conditions, particularly those involving excessive myeloid cell infiltration. Nevertheless, several limitations should be acknowledged. First, this study focused on acute inflammation; whether carnosine influences long-term repair, fibrosis, or epithelial regeneration remains unknown. Second, the downstream molecular pathways by which carnosine regulates macrophage polarization—such as NF-κB, STAT1, or metabolic signaling nodes—require further mechanistic investigation. A limitation of this study is the small sample size, with only n = 2 and n = 3 in two independent experiments, which may limit the statistical power and generalizability of these results. Although the findings provide preliminary insights, further studies with larger sample sizes are needed to validate these effects. In future research, we will further investigate the relationship between carnosine and lung inflammation to confirm our findings and enhance the robustness of the data. Although our study primarily focused on the metabolic and immune regulation in the lungs, it is important to consider the potential role of the gut microbiota in influencing carnosine concentration. Recent studies suggest that the gut microbiota can regulate systemic metabolic processes, including the modulation of metabolites such as carnosine. Although our study did not directly investigate the impact of the gut microbiota on carnosine levels, it is plausible that microbiota-dependent mechanisms could play a role in the observed effects. This is a notable area for future research. To address this, studies involving microbiota composition analysis through 16S rRNA or metagenomic sequencing and using germ-free or antibiotic-treated models will be essential to determine whether the effects of carnosine are influenced, at least in part, by the gut microbiota.

In conclusion, our findings identify carnosine as a protective metabolic mediator in inflammatory lung injury, capable of suppressing immune cell infiltration and reprogramming macrophage activation. This study underscores the utility of metabolomics in discovering endogenous anti-inflammatory regulators and provides a compelling rationale for developing carnosine-based interventions for the treatment of inflammatory lung diseases.

## Data Availability

The original contributions presented in the study are included in the article/Supplementary Material; further inquiries can be directed to the corresponding author.
