# Supplementary material for: Carnosine as a protective metabolic mediator in inflammatory lung injury by inhibiting macrophage infiltration and M1-like polarization
Source: Front Pharmacol. 2025 Nov 21;16:1689575. doi: 10.3389/fphar.2025.1689575 (PMC12679277; doi:10.3389/fphar.2025.1689575)
Supplement: Supplementary file 2 [file Table1.docx]

Table S1

| Categories | Score criteria | | Score  (Total score=27 per lung)^a^ |
| --- | --- | --- | --- |
| General evaluation | Assessment percentage of lung damage area under 4x objective lens | | 0 none  1 focal lung inflammation within one lobe or <30% of total lung cutting areab  2 diffuse lung inflammation within one lobe or involving 30-70% total lung cutting area  3 diffuse lung inflammation involving more than one lobe or 70% total lung cutting area |
| **Bronchioles** | Assessing appearance and severity of peribronchiolar infiltration, intrabronchiolar wall infiltration and bronchiolar epithelial cell death/ desquamation | | 0 none  1 peribronchiolar infiltration  2 bronchiolar epithelial cell death  3 bronchiolar wall infiltration and or severe epithelium desquamation |
| **Alveoli** | Assessing the appearance, scope involved, and severity of alveolar septal infiltration, alveolar space infiltration, alveolar space exudation and hemorrhage | | 0 none  1 only alveolar wall thickening  2 focal area alveolar space infiltration, exudation or hemorrhage involving <30% of lung cutting area  3 diffuse alveolar space infiltration, exudation or hemorrhage involving >70% of lung cutting area |
| **Vasculature^b^** | Severity of vasculature inflammation | 0 none,  1 only perivascular edema and or perivascular infiltration  2 mild infiltration within the vessel wall without endothelium infiltration  3 intensive infiltration into the smooth muscle, or infiltration beneath the endothelium | |

a. Total score = the scores of general assessment × the scores accumulated from individual category assessment
